# Phylogenomic analysis and dynamic evolution of chloroplast genomes of *Clematis nannophylla*

**DOI:** 10.1038/s41598-024-65154-6

**Published:** 2024-07-02

**Authors:** Jinping Qin, Yushou Ma, Ying Liu, Yanlong Wang

**Affiliations:** College of Animal Husbandry and Veterinary Science, Qinghai University, Xining, 810016 Qinghai China

**Keywords:** *C. nannophylla*, Chloroplast genome, Evolution, SSR, Comparative analysis, Phylogenetic analysis, Computational biology and bioinformatics, Genetics, Molecular biology, Plant sciences, Systems biology

## Abstract

*Clematis nannophylla* is a perennial shrub of *Clematis* with ecological, ornamental, and medicinal value, distributed in the arid and semi-arid areas of northwest China. This study successfully determined the chloroplast (cp) genome of *C. nannophylla*, reconstructing a phylogenetic tree of *Clematis*. This cp genome is 159,801 bp in length and has a typical tetrad structure, including a large single-copy, a small single-copy, and a pair of reverse repeats (IRa and IRb). It contains 133 unique genes, including 89 protein-coding, 36 tRNA, and 8 rRNA genes. Additionally, 66 simple repeat sequences, 50 dispersed repeats, and 24 tandem repeats were found; many of the dispersed and tandem repeats were between 20–30 bp and 10–20 bp, respectively, and the abundant repeats were located in the large single copy region. The cp genome was relatively conserved, especially in the IR region, where no inversion or rearrangement was observed, further revealing that the coding regions were more conserved than the noncoding regions. Phylogenetic analysis showed that *C. nannophylla* is more closely related to *C. fruticosa* and *C. songorica*. Our analysis provides reference data for molecular marker development, phylogenetic analysis, population studies, and cp genome processes to better utilise *C. nannophylla*.

## Introduction

*Clematis* are herbaceous or woody vines of the family Ranunculaceae, with several erect shrubs or perennial herbs^1^. *Clematis* has high ornamental and medicinal value and belongs to the Ranunculaceae family^2–4^. *Clematis* is widely distributed worldwide, with approximately 300 species. It is mainly distributed in China, which has the richest plant resources^4^, with more than 100 species^1,5^. *C. nannophylla* is mainly distributed in arid and semi-arid mountain slope environments in Northwest China^6^ and has good stress tolerance. In addition, they possess important pharmaceutical, economic, and ecological properties. However, the systematic classification of *Clematis* poses challenges owing to their intricate nature and extensive morphological variability^4^. Currently, most studies have focused on morphological, physiological, ecological, and pharmacological activities^7–9^, whereas there are few basic molecular studies on germplasm resource identification, genetic breeding, resource conservation, and phylogeny. Furthermore, the chloroplast (cp) genome data of *Clematis* previously tested were submitted directly without detailed analysis, thus limiting our overall understanding of their phylogeny and genome evolution. However, studies on the endemic plant *C. nannophylla* are even fewer in northwestern arid and semi-arid areas of China, limiting the protection and development of this plant species. Therefore, it is imperative to better understand the taxonomic status and predict the future populations of *C. nannophylla* to guide more efficient germplasm resource utilisation, conservation, and breeding strategies.

As the organelles are involved in angiosperm photosynthesis, cps provide energy for plant metabolism. Cps are semi-autonomous genetic organelles that contain a unique genome and gene expression system^10^. Maternal inheritance of cp genomes is prevalent in most angiosperms. Nevertheless, in a minority of instances, it is inherited paternally or through a biparental mode^11^. Compared to the mitochondrial genome, cp genes exhibit greater stability in their genome structure and a heightened rate of evolution. Angiosperm cp genomes are known for their structural and sequence conservation^12^. The cp genome has a distinctive quadripartite structure consisting of a large single-copy (LSC) region and small single-copy (SSC) regions separated by a pair of long inverted repeat (IRa and IRb) regions^10,13^. Although the cp genome is conserved, recent studies have identified many genetic mutations in the cp genome, such as loss of gene or intron fragments, insertion and deletion of bases, changes in the length of reverse repeat regions, insertion/deletion of partial fragments, expansion or deletion of entire reverse repeat regions, and gene rearrangement^14–17^, which may lead to variations in plant structure and adaptation and contribute to plant species identification and future selective breeding^18^.

Furthermore, plant cp genomes encompass a substantial wealth of molecular data, serving as a valuable asset for plant systematics, population genomics, and phylogenetic investigations^19^; for instance, they can be employed in DNA barcoding, research on transplants, and the examination of population-level evolutionary patterns. Moreover, they offer valuable genetic indicators for establishing phylogenetic connections^20–22^. However, the cp genome of *C. nannophylla* has not yet been determined, and a comprehensive analysis of the cp genome structure of *Clematis* genus persists. There are numerous reports on the cp genome of *Clematis*^23–27^. However, few comparative analyses of cp genome structure are available^28^. The *Clematis* cp genomes were used as related species for studying other species^29^. Recent phylogenetic analyses using complete cp genome sequences have provided important insights into two small genera closely related to *Clematis*, *Archiclematis* and *Naravelia*, and have suggested that they should be included in *Clematis*^30^.

Therefore, to establish the taxonomic boundaries and phylogenetic relationships between *C. nannophylla* and other groups, we determined the cp genome characteristics of *C. nannophylla*. This study aimed to (1) obtain the complete sequence of the cp genome of *C. nannophylla* and (2) analyse the phylogenetic positions of 78 coding genes in *C. nannophylla*. (3) The coding and non-coding regions of the cp genome were compared between *C. nannophylla* and three other *Clematis* species, and the effective regions of the cp genome of *C. nannophylla* were determined. (4) Phylogenetic studies of the *Clematis* genus based on the complete cp genome and protein sequences have clarified the phylogenetic relationship and evolution of *C. nannophylla*.

## Materials and methods

### Plant material, DNA extraction, and genome sequencing

Healthy and mature leaves of *C. nannophylla* were sampled from Guide County (36° 7′ 19.92″ N, 101° 35′ 10.68″ E, Altitude: 2192.80 m), Qinghai Province, China, and preserved in liquid nitrogen for further study. Haifeng and Ying used macroscopic botanical identification methods to classify plant materials. Plant specimens were obtained from the College of Animal Science and Veterinary Science, Qinghai University, China, under voucher number QXYTXL220715. The leaves of *C. nannophylla* were conserved in Drikold, delivered to Genesky Biotechnologies Inc. for cp genome extraction and sequencing, and then assembled and further analysed by Genesky Biotechnologies Inc.

### DNA extraction, sequencing, and assembly

#### Sample quality control

Firstly, Nanodrop was used to detect the concentration and purity of the sample, and the concentration was ≥ 20 ng/µL, the total amount was ≥ 100 ng, and OD260/OD280 = 1.8–2.2. The integrity of the DNA samples was tested by agarose gel electrophoresis, which required the main band of genomic DNA to be visible without evident degradation or dispersion.

#### Random DNA library construction

A random sequencing library was constructed using a transposable enzyme library-building kit. The library was constructed quickly and efficiently using transposition enzymes to randomly interrupt DNA and attach splices to both ends of the fragment.

#### PCR amplification of DNA libraries

A high-fidelity polymerase was used to amplify the original library to ensure a sufficient library volume in the sequencer. PCR was used to introduce a specific index and sequencing connectors at both ends of the library. The number of PCR amplification cycles was maintained between 12 and 15. The bias introduced by excessive amplification cycles was reduced to ensure sufficient product yield.

#### Size selection of library fragments

For enlarged libraries, fragment size screening was performed using the Agencourt SPRIselect fragment screening kit while purifying the libraries. A double-sized selection screening method was used in this study. First, the SPRI magnetic beads were used to remove the left side of the target area. The large fragment on the right-side size selection was removed, and a sequencing library with a fragment peak value of 300 bp was screened.

#### Library quality check

The sequencing library was then inspected and quantified. Qubit was used to accurately quantify the library concentration for the accurate mixing of samples to ensure the proper and balanced data volume of each sample. An Agilent 2100 Bioanalyzer was used to determine the size distribution of the library fragments and to evaluate their suitability for computer use.

#### Library pooling and sequencing

Qualified samples were diluted with an equal molar number of samples mixed in the machine. The library was sequenced using an Illumina HiSeq platform (Illumina, USA) with a 2 × 150 double-ended sequencing strategy.

#### Data quality assessment and assembly

Quality assessment of the original sequencing data was performed using FastQC software and R. To ensure high-quality sequencing data and enhance the accuracy of subsequent biological information analysis. The initial data underwent quality control and filtering based on specific criteria: (1) Removal of sequences containing more than 3 N bases; (2) Elimination of sequences with less than 60% high-quality bases (Phred score ≥ 20); (3) Trimming of low-quality bases at the 3′ end; (4) Exclusion of sequences shorter than 60 bp. After quality control, clean reads for *C. nannophylla* totalled 85,520,496 reads. As the sequences may include non-target sequences, they were assembled into contigs using metaSPAdes V3.13.0 software, resulting in 138 Contigs for *C. nannophylla*. Subsequent assembly analysis was conducted against the reference genome *C. florida* (NC058885) to assess contig formation, correct contig orientation, and to determine the starting base position.

### Annotation and analysis of the cp genome sequences

According to the reference species (*Clematis florida*:NC_058885, *Clematis fruticosa*:NC_065273, *Clematis tomentella*:NC_065291, *Clematis songorica*:NC_065290), cps were annotated with CPGAVAS2 software, GenBank files were mapped with CPGView software (http://www.1kmpg.cn/cpgview/), the collinearity between the sample and the corresponding reference genome was analysed using BLAST V 2.9.0 software (https://blast.ncbi.nlm.nih.gov/Blast.cgi), and the collinearity results were analysed using Circos V 0.69-6 software.

SSRs (Simple Sequence Repeats) were analysed using the Perl script MISA V1.0 (https://webblast.ipk-gatersleben.de/misa/index.php), and the minimum number of repeats of mononucleotides, dinucleotides, trinucleotides, tetranucleotides, pentanucleotides, and hexanucleotides was set to 10, 5, 4, 3, 3, and 3, respectively^31,32^. Tandem repeats were identified using the Tandem Repeats Finder v. 4.09 (https://tandem.bu.edu/trf/submit_options)^33^. REPuter software (https://bibiserv.cebitec.uni-bielefeld.de/reputer) identified dispersed repeats, including forward (F), reverse (R), complement (C), and palindromic (P) match repeats, with a minimal length of 8 bp and a Hamming distance of 3^31,34^.

Nucleotides A, T, C, and G were acquired using the CodonW program (version 1.4.2, available at https://sourceforge.net/projects/codonw/)^33^. To assess bias in nucleotide usage within the coding genes of *C. nannophylla*, we employed parity rule 2 (PR2) analysis. Mapping was performed using Origin 2021 Ink^34^.

### Phylogenetic analysis

Combined with 32 previously reported *Clematis* plastomes, we constructed a phylogenetic tree using the newly sequenced *C. nannophylla* complete cp genome and 32 other cp genomes, including one family and two outgroups, downloaded from the NCBI for Biotechnology Information database. MAFFT (v7.313) was used for multiple sequence alignment^35^. Aligned complete cp genome sequence data were utilised to determine the optimal sequence model (ML) using MEGA 11 software, with the GTR + I + G model identified as the best model. Phylogenetic relationships were analysed using MEGA 11 and the Maximum Likelihood (ML) method was used to construct a phylogenetic tree with 1000 bootstraps^33,34^.

### Genome structure comparison

Based on the above results of the phylogenetic analysis, the MVISTA format files of the four *Clematis* species were submitted to an online analysis tool for comparative cp genomes (mVISTA software, http://genome.lbl.gov/vista/mvista/submit.shtml) with the shuffle-LAGAN mode using the annotation of *C. fruticosa* as a reference^34,36^. The analysis of the IR boundaries in four *Clematis* cp genomes involved examining the expansion and contraction using the IRscope tool (https://irscope.shinyapps.io/IRapp/).

### Adaptive evolution and phylogenetic analyses

Based on the cp genomes of *C. nannophylla* and the four other *Clematis* plants in this study, the Ka/Ks values for each functional protein-coding gene and the nucleotide diversity (Pi) values of the four *Clematis* cp genomes were calculated using DnaSP v6.0 software at default settings^37^. The Origin 2021 software was used to plot the data.

### Ethics approval and consent to participate

The sampling of three newly sequenced *C. nannophylla* species was approved by Qinghai province of China and met local policy requirements. Our experimental research, including the collection of plant materials, are complies with institutional, national or international guidelines.

## Results

### Features of the *C. nannophylla* cp genome

**Figure 1:**
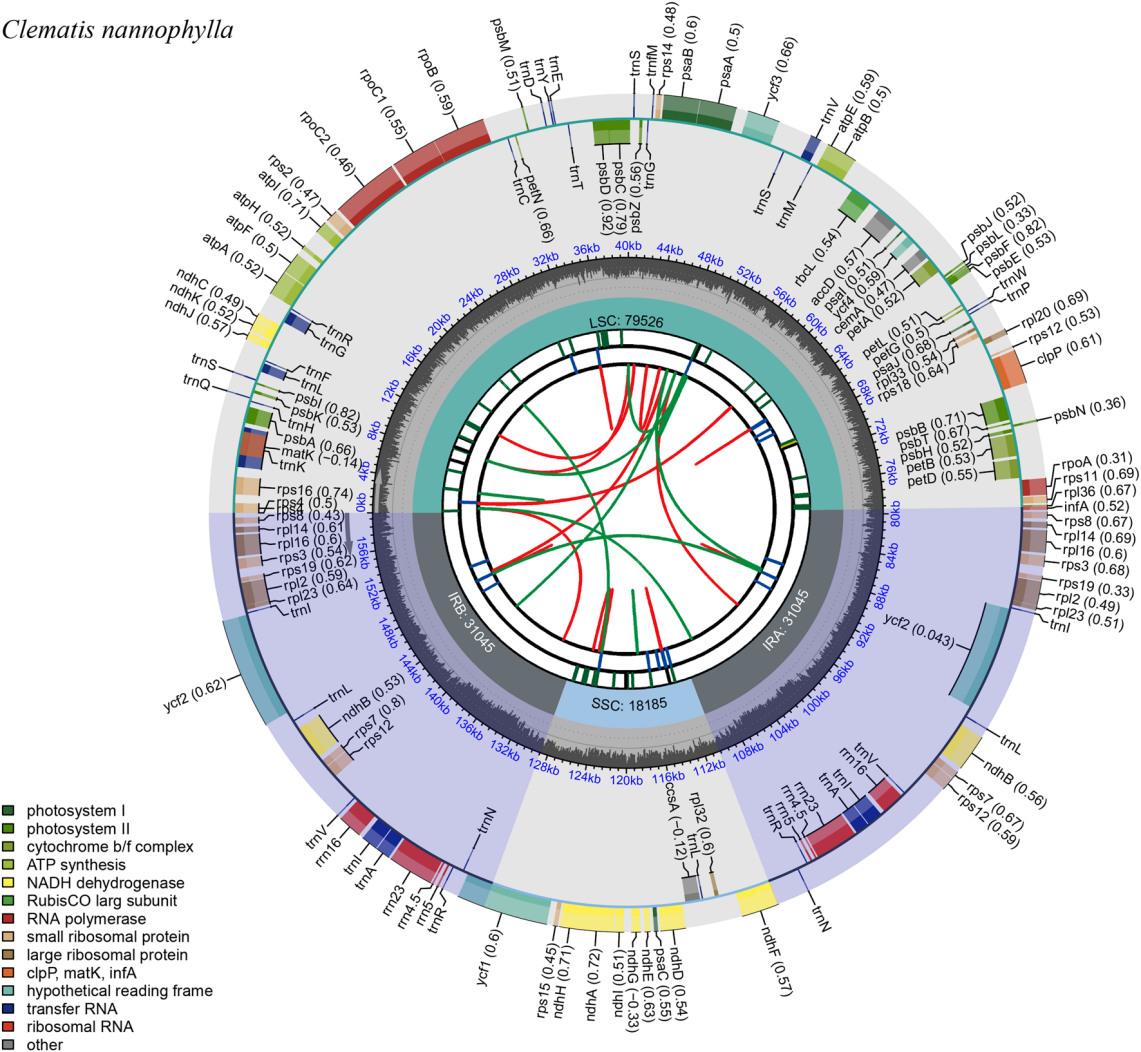
Gene map of the *C. nannophylla* chloroplast genomes. From the center outward, the first track shows the dispersed repeats. The dispersed repeats consist of direct (D) and Palindromic (P) repeats. The second track shows the long tandem repeats as short blue bars. The third track shows the short tandem repeats or microsatellite sequences as short bars with different colors. The GC content along the genome is plotted on the fifth track. The base frequency at each site along the genome will be shown between the fourth and fifth tracks. The genes are shown on the sixth track.

In total, 23,142,846 paired-end reads were obtained from the Illumina NovaSeq platform, with Q20 and Q30 values of 95.0% and 88.3%, respectively. The complete cp genome sequence of *C. nannophylla* was assembled de novo and uploaded to the NCBI for Biotechnology Information database (GenBank accession number: OQ581857). The circular cp genome of *C. nannophylla* is 158,091 bp in size (Fig. 1) and comprises an LSC (79,526 bp) region, two inverted repeat (IR, 31,045 bp) regions, and a small single-copy (SSC, 18,185 bp) region. The highest GC content was observed in the IR region (42.1%), whereas the lowest GC content was observed in the SSC region (31.3%); the average GC content of the whole genome was 38%.

There were 133 predicted functional genes in the *C. nannophylla* cp genome, including 89 protein-coding genes, 36 tRNA, and eight rRNA genes (Tables 1, 2). Protein-coding, tRNA, and rRNA genes accounted for 66.92%, 27.07%, and 6.02% of all annotated genes, respectively. Most genes and protein-coding genes were located in the LSC region, and only 9.02% were located in the SSC region.

**Table 1:** Characteristics of *C. nannophylla* cp genome.

Category	Item	Describe
Chloroplast genome structure	cp gene/bp	159,801
	LSC/bp	79,526
	SSC/bp	18,185
	IRA/IRB/bp	31,045
	CDS/bp	80,652
Gene composition	cp gene	133
	CDS	89
	tRNA	36
	rRNA	8
GC content	cp gene	38
	LSC	36.3
	SSC	31.3
	IRA/IRB	42.1
	CDS	38.4

**Table 2:** Genes in cp genome of *C. nannophylla*.

Category	Gene group	Gene name
Photosynthesis	Subunits of photosystem I	*psaA*, *psaB*, *psaC*, *psaI*, *psaJ*
	Subunits of photosystem II	*psbA*, *psbB*, *psbC*, *psbD*, *psbE*, *psbF*, *psbH*, *psbI,psbJ*, *psbK*, *psbL*, *psbM*, *psbN*, *psbT*, *psbZ*
	Subunits of NADH dehydrogenase	ndhA*, ndhB* (2), *ndhC*, *ndhD*, *ndhE*, *ndhF,ndhG*, *ndhH*, *ndhI*, *ndhJ*, *ndhK*
	Subunits of cytochrome b/f complex	*petA*, petB*, petD*, *petG*, *petL*, *petN*
	Subunits of ATP synthase	*atpA*, *atpB*, *atpE*, atpF*, *atpH*, *atpI*
	Large subunit of rubisco	*rbcL*
	Subunits photochlorophyllide reductase	–
Self-replication	Proteins of large ribosomal subunit	*# rpl22*, *rpl14(2)*, rpl16*(2), rpl2* (2), *rpl20*, *rpl23 (2)*, *rpl32*, *rpl33*, *rpl36*
	Proteins of small ribosomal subunit	# rps16*, *rps11*, rps12* *(2)*, *rps14*, *rps15*, *rps18*, *rps19(2)*, *rps2*, *rps3(2)*, *rps4*, *rps7 (2)*, *rps8(2)*
	Subunits of RNA polymerase	*rpoA*, *rpoB*, rpoC1*, *rpoC2*
	Ribosomal RNAs	*rrn16 (2)*, *rrn23 (2)*, *rrn4.5 (2)*, *rrn5 (2)*
	Transfer RNAs	trnA-UGC* *(2)*, *trnC-GCA*, *trnD-GUC,trnE-UUC*, *trnF-GAA*, *trnG-GCC*, trnG-UCC*,trnH-GUG, *trnI-CAU (2)*, trnI-GAU* (2),trnK-UUU*, *trnL-CAA (2)*, trnL-UAA*,trnL-UAG, *trnM-CAU*, *trnN-GUU (2)*, *trnP-UGG*, *trnQ-UUG*, *trnR-ACG (2)*, *trnR-UCU*, *trnS-GCU,trnS-GGA*, *trnS-UGA*, *trnT-GGU*, *trnV-GAC (2)*, trnV-UAC*, *trnW-CCA,trnY-GUA*, *trnfM-CAU*
Other genes	Maturase	*matK*
	Protease	clpP**
	Envelope membrane protein	*cemA*
	Acetyl-CoA carboxylase	*accD*
	c-type cytochrome synthesis gene	*ccsA*
	Translation initiation factor	*# infA*
	Other	–
Genes of unknown function	Conserved hypothetical chloroplast ORF	*# ycf1*, *ycf2 (2)*, ycf3**, *ycf4*

^#^ Gene, Pseudo gene; Gene (2), Multiple copy gene, the number of copies in parenthesis; Gene*, Gene with one intron; Gene**, Genes containing two introns.

Subsequently, we annotated all the assembled genes and their functions. These genes belong to four types: photosynthesis-related, self-replication-related, genes with unknown functions, maturases (*matK*), and proteases (*clpP*). A total of 22 annotated genes were double-copy genes, including 11 protein-coding genes, seven tRNAs, and four rRNAs. Sixteen genes (*atpF*, *ndhA*, *ndhB*, *petB*, *petD*, *rpl16*, *rpl2*, *rpoC1*, *rps12*, *rps16*, *trnA-UGC*, *trnG-UCC*, *trnI-GAU*, *trnK-UUU*, *trnL-UAA* and *trnV-UAC*) each contained one intron, whereas the protein-coding genes *ycf3* and *clpP* contained two introns (Table 2). The longest intron (2,554 bp) was found in *tunK-UUU*, which completely encompassed matK, and the smallest intron (492 bp) was found in *trnL-UAA*.

**Figure 2:**
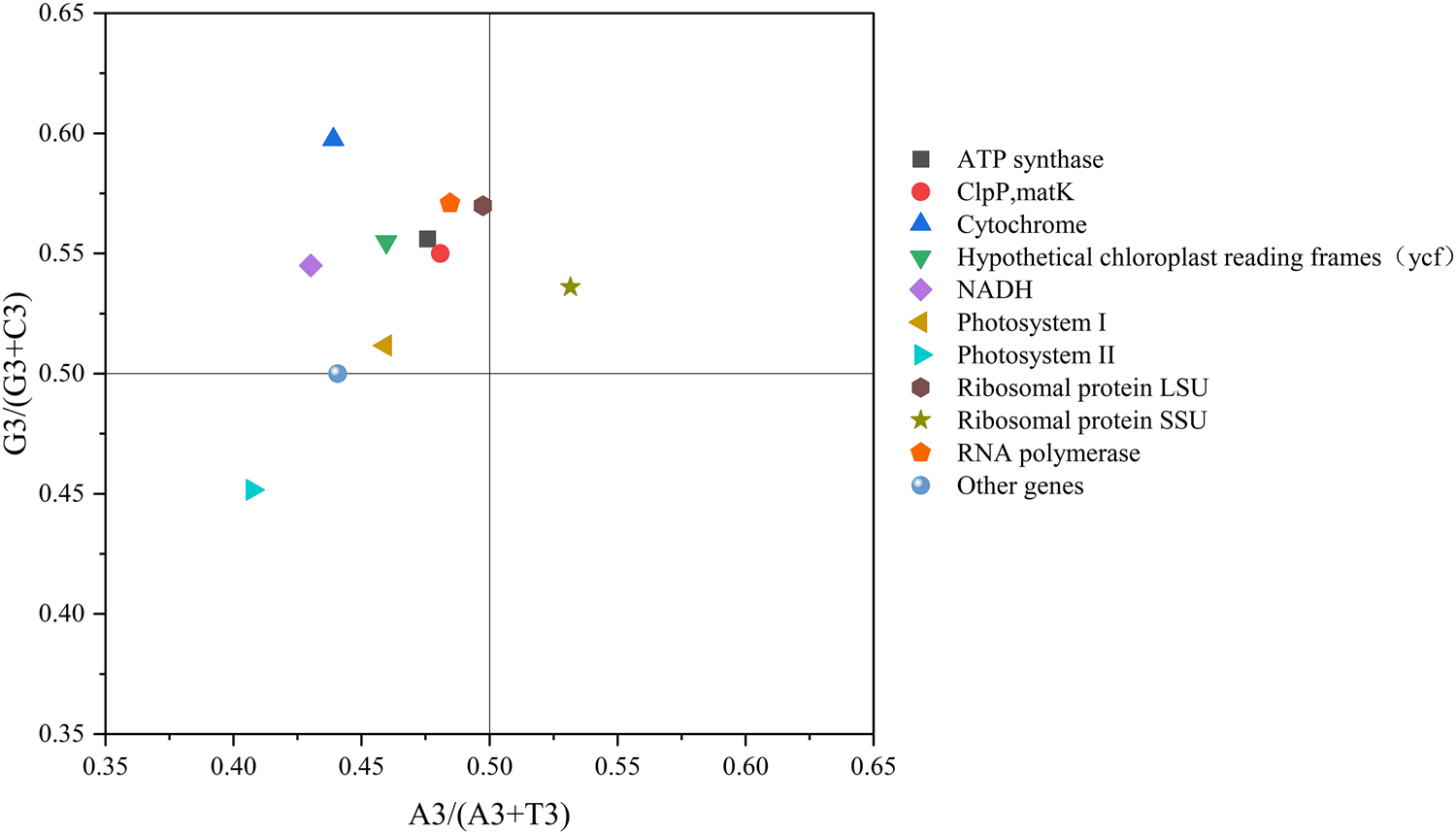
PR2 analysis for genes in the cp genome of *C. nannophylla*. A3, T3, C3, and G3 represent nucleotide A, T, C, and G content at the third position of synonymous codons.

PR2 plot mapping analysis was performed using the protein-coding gene sequences of *C. nannophylla* (Fig. 2), which were constructed to show the relationship between A3/(A3 + T3) and G3/(G3 + C3), and the data were distributed into four quadrants in a scatter diagram. Most genes were located in the second quadrant, with ribosomal protein SSU genes in the first quadrant (G > C, A > T) and photosystem II genes in the third quadrant (C > G, T > A).

### Codon usage bias

**Figure 3:**
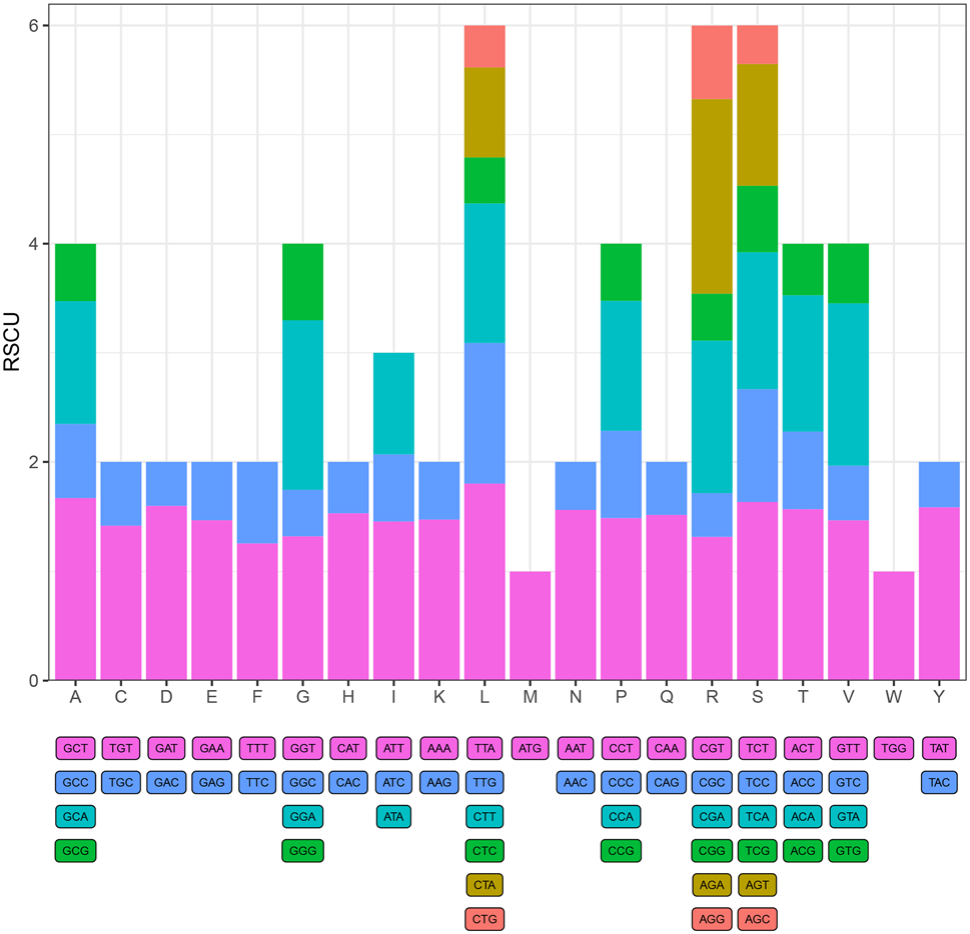
Counting of relative synonymous codon usage (RSCU) of amino acids in the cp genome of *C. nannophylla*. The colors of the histogram correspond to the colors of the codons.

As each amino acid corresponds to at least one or up to six codons, codon use varies widely among organisms and species^38^, and this difference in synonymous codon usage is referred to as codon preference. Natural selection, species mutations, and genetic drift may cause bias in codon use. We selected a codon bias unique to the CDS genome, and the results showed that 26,795 amino acids were detected in the cp genome of *C. nannophylla* (Fig. 3), of which leucine was the most abundant with 2744 codons (10.2%), followed by isoleucine with 2350 codons (8.8%), serine and glycine with 2070 and 1851 codons (7.7% and 6.9%, respectively), and cysteine was the least abundant, with 214 codons (1.2%) and 30 (49.18%) preferred codons (RSCU > 1). Methionine and tryptophan had RSCU values equal to 1, but the preferred codon was TTA, which encodes leucine (Leu) with an RSCU value of 1.806.

### Detection of cp genome repeat sequences and SSRs

**Figure 4:**
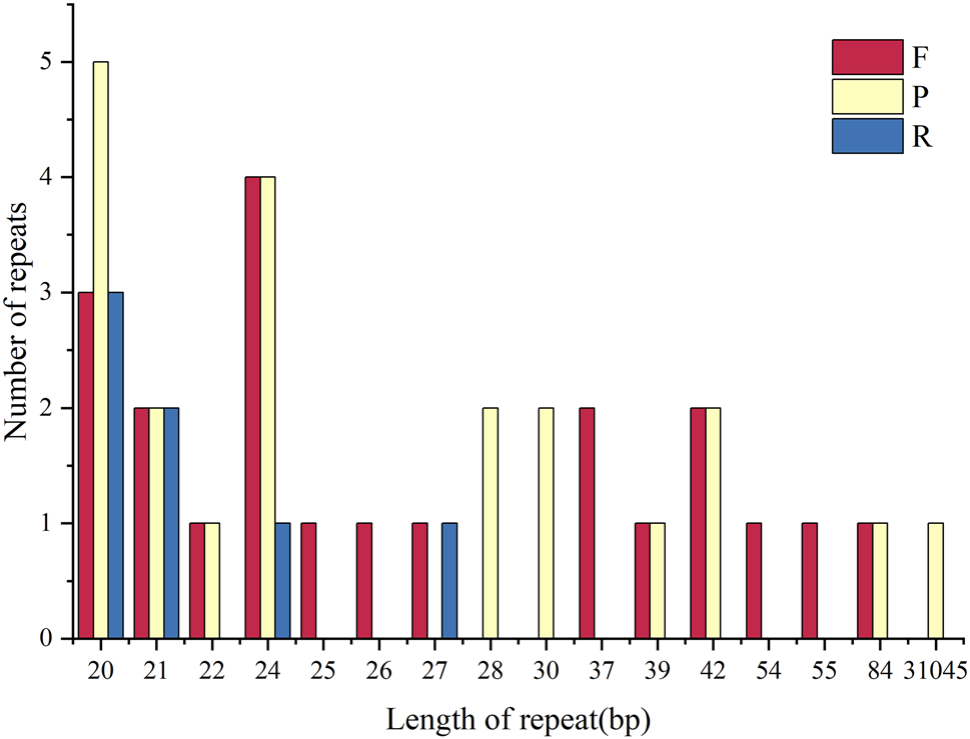
Repeated sequences of the *C. nannophylla* cp genome. The abscissa is the length of the repeat sequence, and the ordinate is the number of repeat sequences. F stands for forward repetition, P for palindromic repetition, R for reverse repetition, and C for complementary repetition.

The abscissa represents SSR repetition units, and the ordinate represents the number of SSRs of each type. To learn the repeat sequence of the *Clematis* cp genome, four categories of repeat sequences were detected and analysed. There were no complementary repeats in *Clematis* (Fig. 4), and the number of repeats was highest in *C. songorica* (75) and lowest in *C. florida* (71). The number of discrete replicates of *C. nannophylla* was 74, second only to *C. songorica*. Forward, palindromic, and tandem repeats were most common. A total of 50 dispersed repeats were found in the *C. nannophylla* cp genome, including 22 forward, 21 palindromic, and seven reverse repeats, which were more than 20 bp in length in *C. nannophylla*, which is different from other *Clematis* species. The most dispersed and tandem repeats were 20–30 bp and 10–20 bp, respectively.

**Figure 5:**
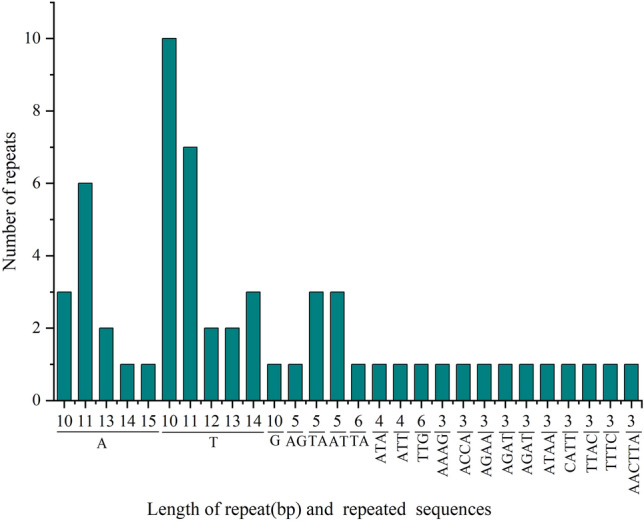
Counting of the types of SSRs.

We detected 66 SSRs in the *C. nannophylla* cp genome using the MISA Perl script (Fig. 5). The SSRs were mainly distributed in the LSC region (45, 68.18%), followed by the IR region (15, 22.73%). Additionally, 49 SSRs were located in intergenic spaces, and 17 SSRs were located in genes, such as *matK*, *psbC*, *rpoB*, *rpoC2*, *clpP*, *petB*, *rps3*, *ndhA*, *trnV-UAC*, *rpl16*, and *ycf1*. The SSRs consisted of 39 mononucleotides, eight dinucleotides, three trinucleotides, nine tetranucleotides, one hexanucleotide, and six complex nucleotide repeats. Moreover, oligo A and T repetitions accounted for 21.21% and 36.36% of the total SSRs, respectively, whereas oligo C and G repetitions were uncommon, and only one mononucleotide (G10) was detected in *C. nannophylla.*

### Comparison of complete Cp genomes

**Figure 6:**
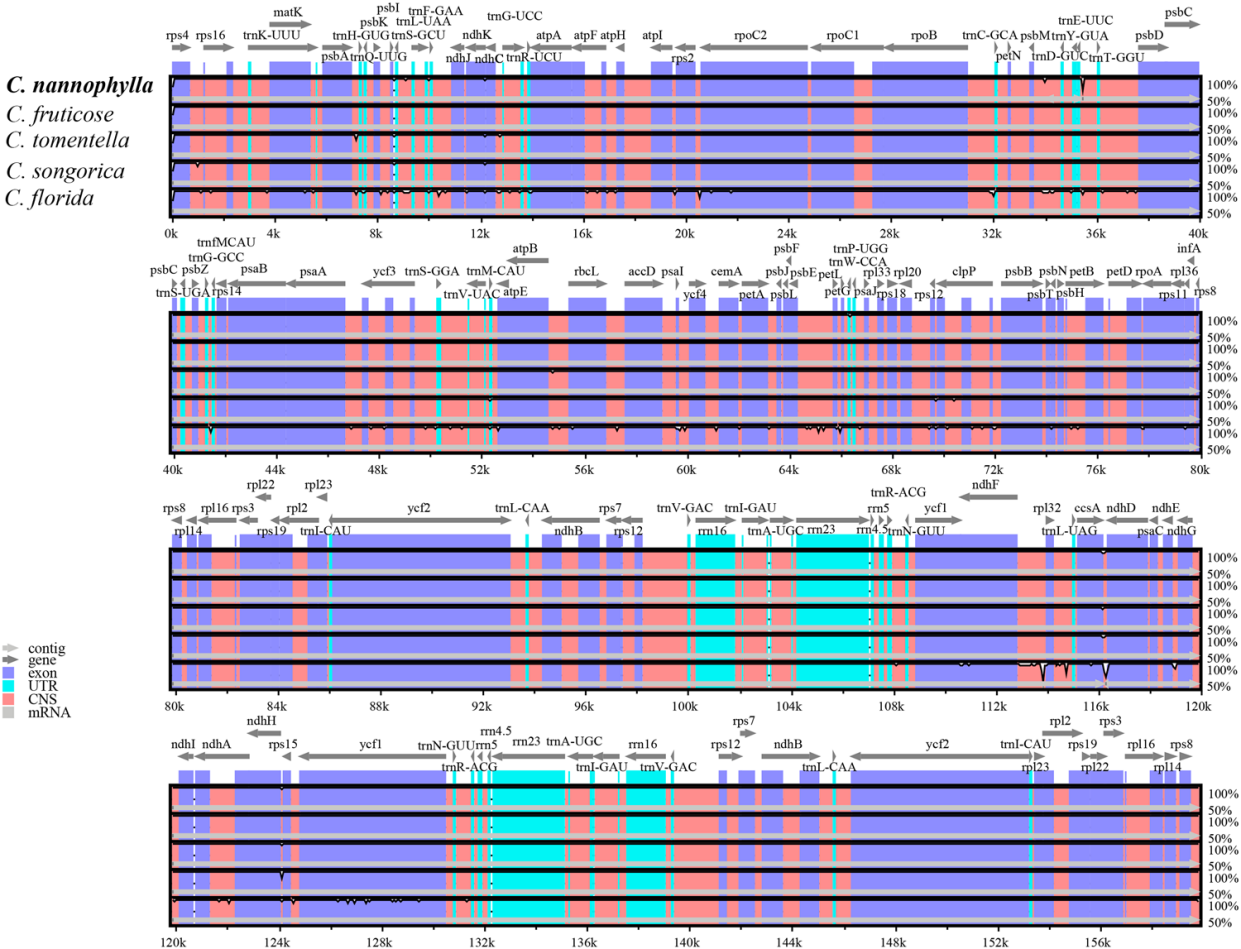
Sequence alignment of *C. nannophylla* chloroplast genomes. With *C. florida* as a reference. The y-axis indicates the percent identity between 50 and 100%. Genome regions colored represent protein coding regions, rRNA coding regions, tRNA coding regions or conserved noncoding sequences (CNS). White peaks indicate the regions with sequence variation among *Clematis* species.

The cp genome sequences of *C. nannophylla* were analysed using the *BLAST* program on the *NCBI* Biotechnology Information website (http://www.ncbi.nlm.nih.gov/blast). The *C. fruticosa* plant, which is most similar to *C. nannophylla,* was selected for this study (Fig. 6). Therefore, the complete cp genomes of the five *Clematis* species were represented using the mVISTA program with *C. fruticosa* as the reference.

The results showed that the cp genome of *Clematis* was highly conserved and that the LSC and SSC regions were more divergent than the IR regions. Furthermore, the coding regions were more conserved than the non-coding regions in our alignment, and the differences between *C. nannophylla* and *C. fruticosa* were not statistically significant. There was only one evident difference between *trnE-UUC*-*trnT-GGU*. However, there were many divergent regions in *C. florida*. These divergent regions mainly included *psbA*-*atpA*, *atpI*-*rpoC2*, *rpoB*-*psbD*, *psbE*-*petG*, *clpP*, and *rpoC2*, most of which were found in the intergenic regions. The most divergent coding regions were *clpP* and *rpoC2*, known as hotspot regions, because they contain variations such as single-nucleotide polymorphisms and indels, which can be used as molecular markers in DNA barcoding and phylogenetic analysis of *C. nannophylla*.

### IR expansion and contraction

**Figure 7:**
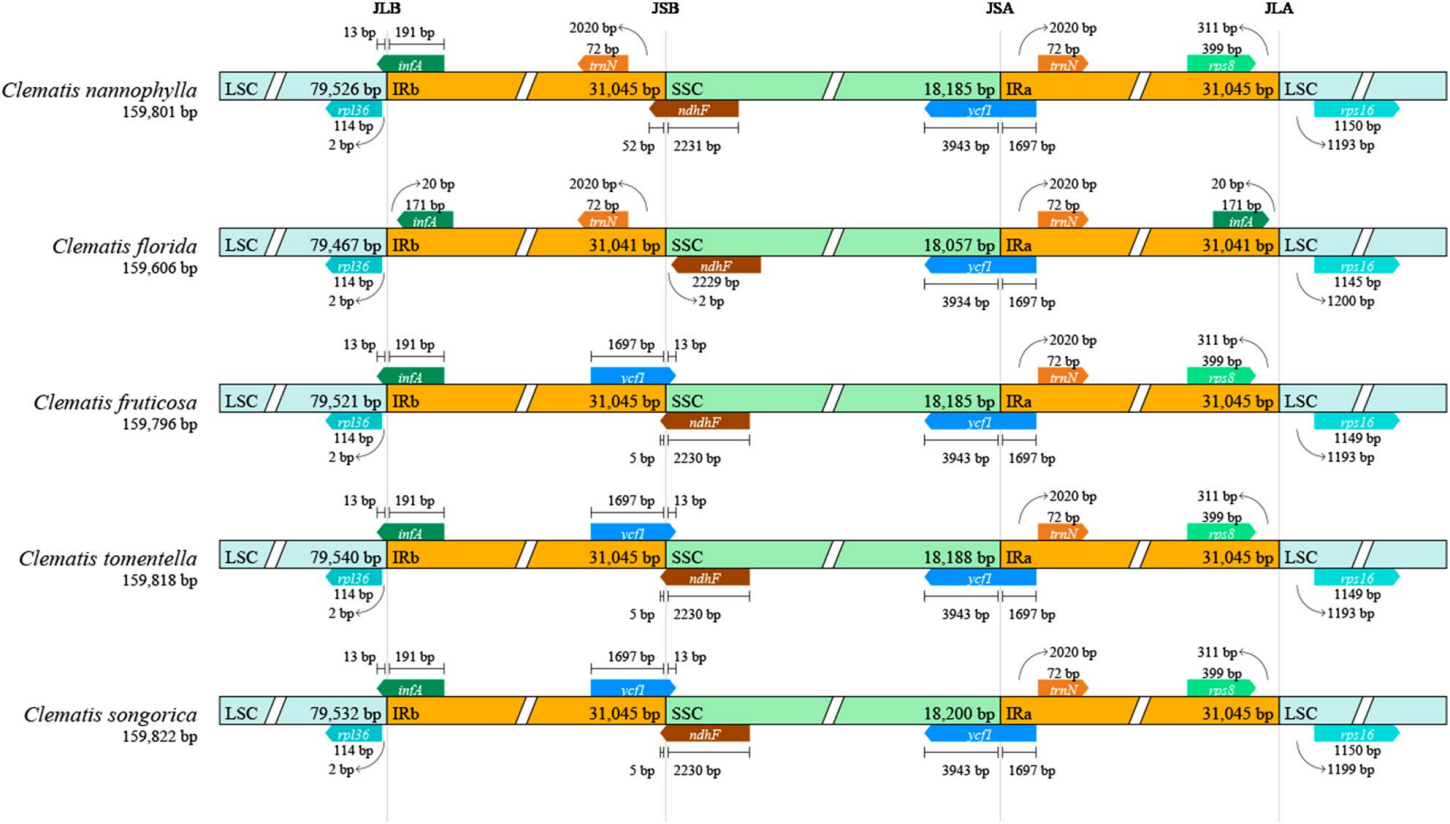
Comparisons of LSC, SSC, and IR border regions in the chloroplast genome of five *Clematis* species.

As a highly conserved region of the cp genome, the expansion and contraction characteristics of the IR region are mainly responsible for changes in cp genome size and rearrangement. Therefore, to compare IR expansion and contraction in the cp genome of *C. nannophylla* with those of the four *Clematis* plants, we analysed the border structure of *C. nannophylla* and the four reference *Clematis* cp genomes (Fig. 7).

The genes located in the binding regions of LSC/IRb, IRb/SSC, SSC/IRa, and IRa/LSC were *rpl36*, *infA*, *ycf1*, *trnN*, *ndhF*, *ycf1*, *trnN*, *rps8*, and *rps16*. The *rpl36* and *infA* genes were located at the junctions of the LSC/IRb border. The *rpl36* gene was located in the LSC region, and the *infA* gene of *C. florida* was located exclusively in the IR region, 20 bp away from the LSC/IRb border, whereas those of other *Clematis* species extended into the LSC regions.

*trnN* was completely located in the IRb region of *C.nannophylla* and *C. florida* and 72 bp from the IRb/SSC boundary. However, the *ycf1* gene was found at the IRb/SSC boundary of the other three *Clematis* species (*C. fruticose*, *C. tomentella*, and *C. songorica*), which extended into the SSC regions; IRb/SSC extended into ndhF genes in all *Clematis* species, except for C. *florida*.

The distribution of *ycf1* and *trnN* at the SSC/IRa boundary was the same in all five *Clematis* species. All *ycf1* genes were embedded at the SSC/IRa border, with 3943 and 1697 bp located in the SSC and IRa regions, respectively. The *trnN* genes are all located in the IRa region, 72 bp away from the SSC/IRa boundary.

Except for C. *florida*, *rps8* genes completely located in the IRa region were 311 bp away from the IRa/LSC boundary, whereas *infA* genes were found in *C. florida* completely located in the IRa region, 20 bp away from the IRa/LSC boundary. The distance between *rps16* and the IRa/LSC boundary in the five *Clematis* species ranged from 1,193 to 1,200 bp. Based on these results, The IR, LSC, and SSC regions of *C. nannophylla* were found to be slightly different from those of the other four *Clematis* species at the boundary, and the numbers and sequences of the genes in these regions were conserved.

### Adaptive evolution analysis

**Figure 8:**
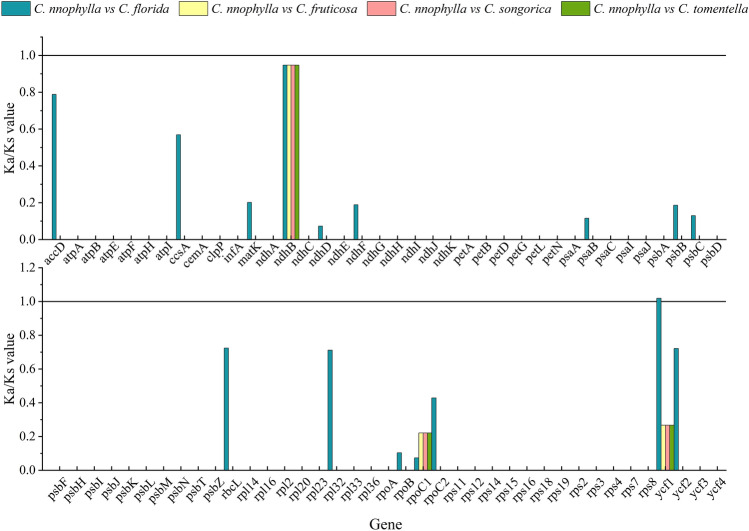
The 79 protein-coding genes of the *C. nannophylla* cp genome and four clematis species were used for Ka/Ks analysis.

Using *C. nannophylla* as a reference, the selection patterns of protein-coding genes were determined by examining synonymous and non-synonymous substitutions in the cp genomes of five *Clematis* species. The Ka/Ks ratios of 78 protein-encoding genes were compared among the five cp genomes (Fig. 8). The Ka/Ks values of most coding genes were less than 1 or 0, which could not be calculated, indicating that they were relatively conserved. In particular, all genes of *Clematis* species had Ka/Ks values less than 1, except *C. florida.* However, the Ka/Ks values of *ycf1* in *C. nannophylla* and *C. florida* were greater than one. The Ka/Ks ratios of *ndhB*, *rpoCl*, and *ycf1* in *C. nannophylla* were similar to those of *C. fruticosa*, *C. songorica,* and *C. tomentella*.

**Figure 9:**
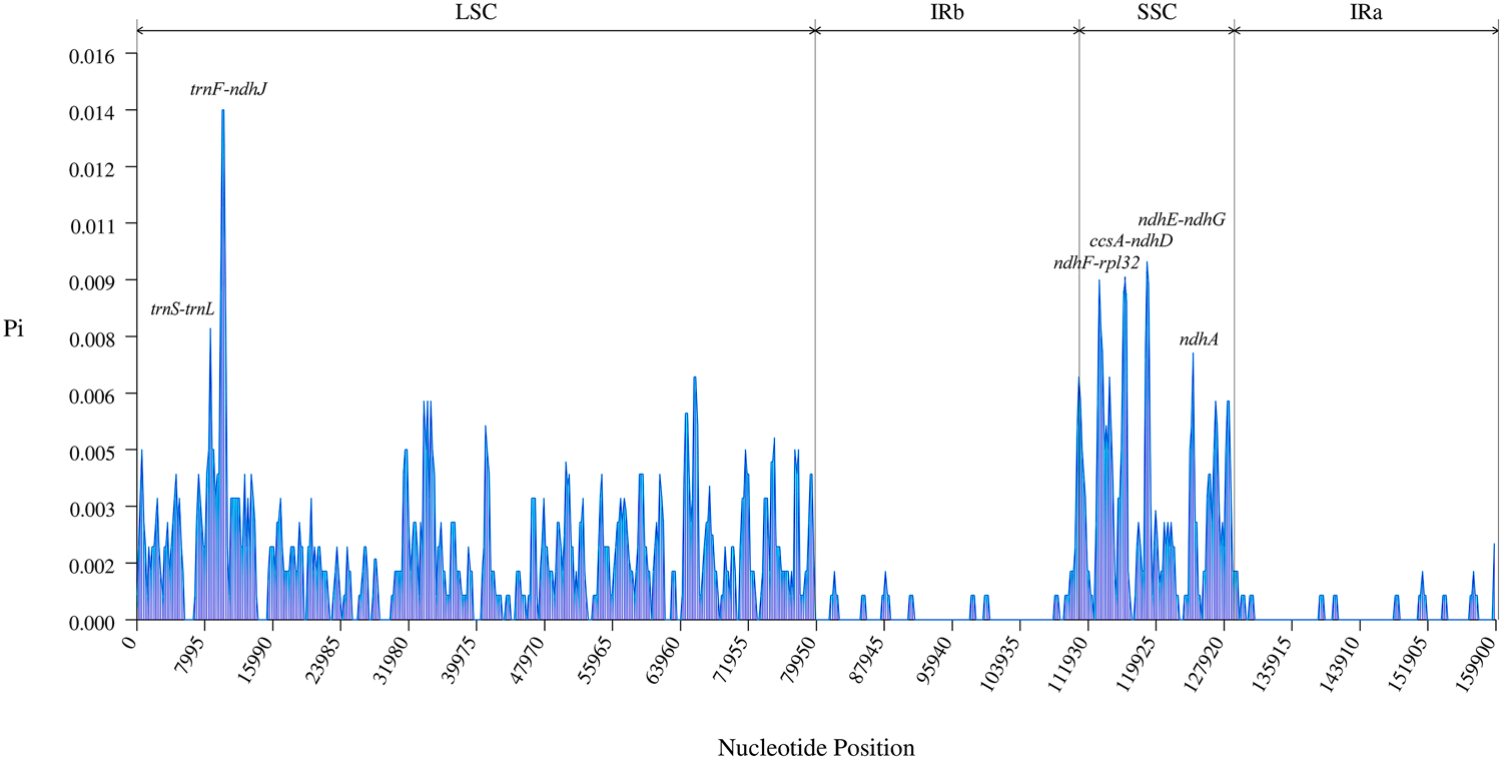
Comparative analysis nucleotide diversity based on the five clematis. x-axis: gene name, y-axis: Pi value.

The nucleotide diversity (Pi) values of the cp genomes of *C. nannophylla* and four other *Clematis* plants (*C. fruticose*, *C. tomentella*, *C. songorica,* and *C. florida*) were calculated to determine the divergent hotspots (Fig. 9). The Pi values within 600 bp of the five *Clematis* cp genomes were calculated. The minimum and maximum values for the entire genome sequence ranged from 0 to 0.014, with an average value of 0.001416.

However, some highly variable loci, including *trnF-ndhJ*, *ndhE-ndhG*, *ndhF-rpl32*, *ccsA-ndhD*, *ccsA, ndhD, trnS-trnL, ndhF-rpl32, rps15*, and *ndhE*, were located more precisely. All these regions had much higher values than the other regions (Pi > 0.007), and most of these higher-value regions were located in the SSC region. In the LSC region, there were a few loci with Pi values greater than 0.007, whereas the IR region had the lowest Pi value. All Pi values were less than 0.003, indicating that the IR regions are substantially more conserved. Based on these results, we believe that r*pl32, ccsA, ndhD, rps15,* and *ndhE,* which have relatively high sequence deviations, are good candidates for interspecies phylogenetic analysis.

### Phylogenetic analysis

**Figure 10:**
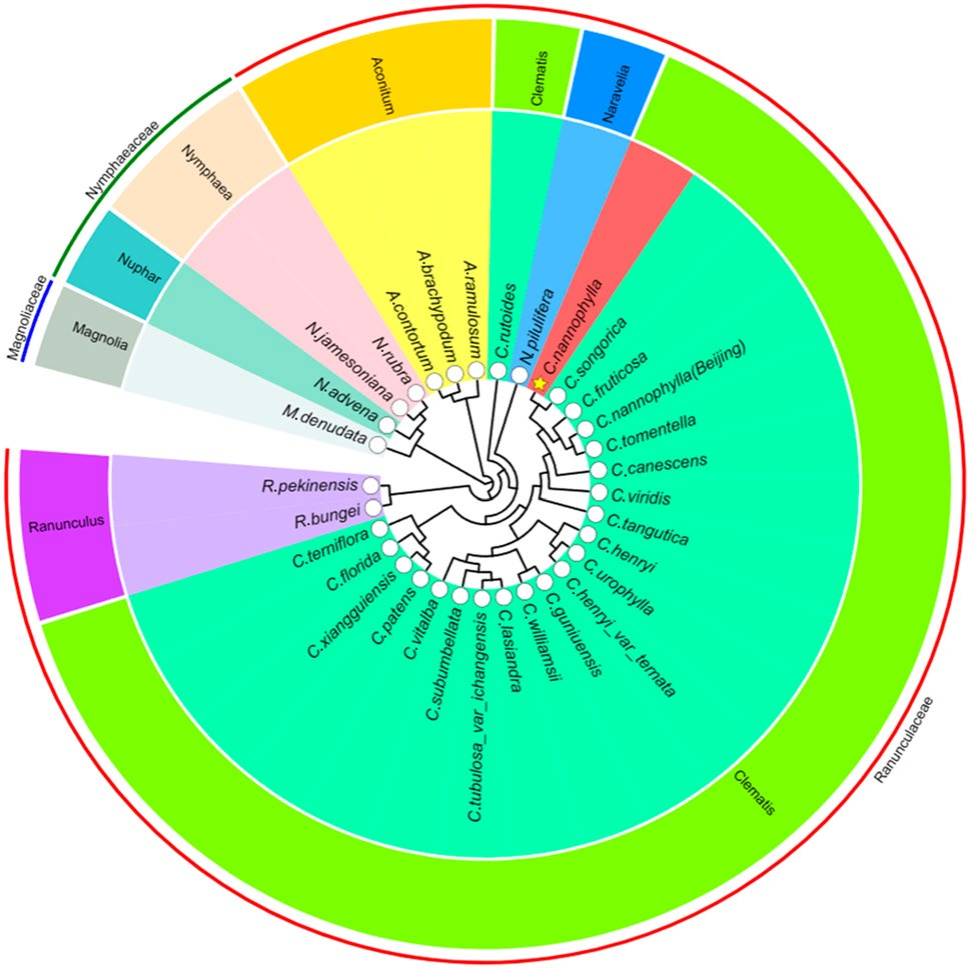
Molecular phylogenetic tree of 32 plants including 22 *Clematis* species based on complete chloroplast genomes. The tree was constructed by maximum likelihood analysis using MEGA11 with a bootstrap test of 1000 replications.

Cp genomes play a significant role in the phylogenetic relationships and evolutionary histories of plants. To determine the phylogenetic position of *C. nannophylla* within Ranunculaceae, a phylogenetic tree was constructed using the best-fit model, GTR + G + I (Fig. 10). The analysis included the complete cp genomes of 17 *Clematis* species and six outgroup genera (three *Aconitum* species, two *Nymphaea* species, two *Ranunculus* species, one *Naravelia* species, one *Nuphar* species, and one *Magnolia* species). The resulting phylogenetic tree consisted of 30 nodes, with 29 nodes having a bootstrap support value of ≥ 80% and 26 nodes having a support value of 90%, indicating the high reliability of the clustering results. The 21 plant species were divided into two large and seven small groups. Magnoliaceae and Nymphaeaceae formed one large group, whereas 28 Ranunculaceae species formed another group, with subgroups including *Clematis*, *Ranunculus*, and *Aconitum*. The analysis revealed that *C. nannophylla* shares high homology with *C. fruticose* and *C. songorica* but shows a distant relationship with other *Clematis* species. Additionally, within the family Ranunculaceae, *Clematis* and *Aconitum* were identified as highly credible sister groups.

## Discussion

### Cp genome structure and size of *C. nannophylla*

Cps are important organelles for photosynthesis and energy production and are essential for plant growth and development^10^. Cps have a unique genome and gene expression system that play a crucial role in metabolism as a source of energy that supports plant life^39^. The complete *C. nannophylla* cp genome showed great similarities to most angiosperms in terms of GC content and quadripartite architecture, including two inverted repeats (IRs), an LSC region and a small SSC, which is common in plants^23,28,39^.

Furthermore, the cp genome of *C. nannophylla* contained 133 genes (including 89 protein-coding genes, 36 tRNAs, and eight rRNAs), and the GC% content of the genome was 38%. High GC content often correlates with earlier phylogenetic differentiation (such as *Nymphaeaceae* and *Magnoliaceae*)^40^. Generally, the complete cp genome of *C. nannophylla* is very similar to other reported cp genomes of *Clematis* plants in terms of length, structure, and gene composition^23,28,41^. There was no evidence of rearrangement, and good collinearity was observed. Aligning the entire cp genome revealed that *C. nannophylla* cp genomes were relatively well conserved; therefore, we concluded that *C. nannophylla* differentiated earlier among Ranunculaceae.

### Cp genome repeat sequence of *C. nannophylla*

Plants contain numerous replicates in their genomes. However, the number, size, type, and location of repeats among different plants^34^ and repeats in the cp genome have been widely used to identify mutation hotspots and determine plant evolutionary relationships^42^. Fifty dispersed repeats were found in *C. nannophylla,* including 22 forward, seven reverse, and 21 palindromic repeats. The number of dispersed repeats was the same as that in other species of *Clematis,* and most of these dispersed repeats were in the LSC region. Most dispersed repeats were 20–30 bp long, indicating that short repeats occurred more frequently than long repeats among the dispersed repeats of *C. nannophylla*. Tandem repeats are generally considered the primary cause of genomic rearrangement and expansions^43^. Tandem repeats of *C. nannophylla* range from 10 to 20 bp, with most tandem repeats located in intergenic spaces or intron regions and a few in the same gene region, *ycf2*^44^.

Simple sequence repeats (SSR) usually consist of 1–6 nucleotide repeating units and have been recognised as important molecular markers for studying population variation^45,46^. As genetic information in the cp genome is inherited only from maternal progenitors, SSR in the cp genome are sensitive to population genetic effects^47^ and have been widely used in the study of population evolution and polymorphism^48^. SSR varied in number and type according to species; 66 SSR repeats were screened in *C. nannophylla,* and their distribution was mainly found in the LSC and SSC regions. The number of variation sites in the IR region is reduced, mainly in the single-copy region^49^. Among the mononucleotide SSR repeats, the number of A/T mononucleotide repeats was significantly higher than that of G/C mononucleotide repeats. This pattern also exists in other angiosperms^44,50^. The dispersed tandem and SSR repeats identified above are responsible for cp genome rearrangement, gene replication, and gene expression; play a vital role in genomic rearrangement and sequence variation in cp genomes; and are helpful for phylogenetic studies. Rearrangements or sequence variations in these repeat units may also lead to substitutions, insertions, and deletions in the cp genome^17,51,52^. These repeat sequences have also been shown to be a source of information for the development of markers that play an important role in population and phylogenetic studies^44^ and can be used for future genetic structure, differentiation, and species identification of *C. nannophylla*. Therefore, they are a source of information for the development of markers and play an important role in population and phylogenetic studies^44^ for future genetic structure, differentiation, and species identification of *C. nannophylla*.

### Codon usage bias in the cp genome of *C. nannophylla*

Codon usage bias is an important feature of genomic evolution and is of great significance in the study of molecular and exogenous gene expression^53^. PR2 further confirmed that most genes in *C. nannophylla* favour T and G in the coding chain rather than A and C and that the direct cause of this base asymmetry is the replication mechanism. However, asymmetry between coding and non-coding strands is an important cause of nucleotide skew^54^. However, the influence of replication mechanisms on base bias differed between the AT and CG asymmetries. Replication is generally strong for GC skew, whereas AT skew is caused by coding sequence-related mechanisms^54,55^.

Codon usage patterns are evolutionary features of the genome. In plants, codon usage bias is related to gene expression and is mainly affected by natural selection and mutation pressure, with differences between species^56^. In the cp genome of *C. nannophylla*, there are 30 high-frequency codons (RSCU > 1); leucine is the most important amino acid, and cysteine is the least important, consistent with the codons observed in other higher plants^53,57,58^. The use of synonymous codons is not random, and the analysis of codon preferences can provide valuable information for understanding species adaptation and molecular evolution.

### Comparative genomic analysis of the cp genome of *C. nannophylla*

The IR regions of angiosperm cp genomes are highly conserved. The expansion and contraction of the IR region boundaries are common evolutionary events in most angiosperms that may lead to variations in cp genome length, gene replication or reduction, and the origin of pseudogenes^59,60^. This study found that the IR expansion and contraction of *C. nannophylla* showed great similarity with those other plants of *Clematis*, and these regional genotypes and distribution locations are similar^38^. However, only minor differences are observed near the IRb/SSC boundaries. *trnN* was not *ycf1* at the IRb/SSC boundary of *C. nannophylla* and *C. florida*, and *infA* was not observed near the IRa/LSC boundary, possibly the result of contraction and expansion of the IR region; this is also an important reason for the differences in cp genome length^33^. The *infA* gene is transcribed as a polycistronic mRNA, a component of the ribosomal protein (*rpl23*) operon, while the *ycf1* gene is a functional gene that encodes essential products for cell survival^61^. Therefore, the loss (or pseudogenisation) of *infA* and *ycf1* may have resulted from gene transfer to the nucleus. However, there is no evidence suggesting that *infA* and *ycf1* are transferred from the cp genome to the nuclear genome of *Clematis*. Further studies on the transcriptomes of these two genes are required to elucidate the effects of length variation on *Clematis*.

Owing to the highly conserved structure and nucleotide content of cp genomes, mutation hotspots of cp genomes can be quickly and accurately identified by comparative analysis. Therefore, mutation hotspots are often used as a basis for highly variable markers (DNA barcodes) in population genetics and phylogenetic studies^62,63^. In this study, we compared the cp genome structures of five *Clematis* species using mVISTA (with *C. fruticosa* as a reference) and found that the non-coding region was more prone to mutations than the coding region. Furthermore, variation in the SC region was higher than that in the IR region, similar to the results of previous plant studies^28,62,64^. *psbA*-*atpA*, *atpI*-*rpoC2*, *rpoB*-*psbD*, *psbE*-*petG*, *clpP*, and *rpoC2* were the most highly variable regions detected in *C. nannophylla*. To determine the degree of variation in these highly variable regions in *C. nannophylla*, nucleotide variability in DNASP v6 was used to identify differences among the cp genomes of *Clematis* and mutation hotspots. Nucleotide diversity (Pi) indicates the degree of variation in the nucleic acid sequences of each species, and sites with high variability can be selected as molecular markers for population genetics^33,65^. In the present study, the nucleotide diversity analysis showed that the gene sequences in the LSC and SSC regions were more variable than those in the IR regions, which is consistent with the results found in Asteraceae and Fagaceae plants^33,66^.

By analysing the cp genome sequence variation of five *Clematis* species, we identified 13 hypervariable regions (Pi > 0.006) in the LSC and SSC regions, which is of great significance for the study of molecular barcodes. Highly variable regions, such as *ndhF*, *ccsA*, and *ndhD*, have also been found in two Korean endemic *Clematis* species^28^. Simultaneously, the same highly variable regions, *ccsA* and *rpl32*, were found in *Fagus longipetiolata* of Fagaceae. The *ccsA* gene is also considered the locus for understanding cp genome evolution in *Fagus longipetiolata* of Fagaceae^33^, *Litsea*^65^, *Pterocarpus*^62^, and *Prosopis genera*^67^. Furthermore, the Pi values of the 13 height-variable regions in this study were greater than 0.006, corresponding to the height-variable regions. Overall, these highly diverse regions provide a wealth of information for the development of molecular markers for the identification of *Clematis* species as well as for the analysis of the phylogenetic relationships and population genetics of *C. nannophylla*.

### Adaptive evolution analysis of the cp genome of *C. nannophylla*

By comparing *C. nannophylla* with four other species of *Clematis*, we detected the protein-coding region genes in *C. nannophylla* under selection pressure. Ka/Ks is generally used to express the selection pressure on protein-coding genes^68^. When Ka/Ks was greater than 1, it indicated a positive selection effect, and when Ka/Ks was less than 1, it indicated a purification selection effect^69^. In this study, the Ka/Ks values of most genes in *C. nannophylla* were less than 1 compared to those of the other four plants, indicating that purification selection played an important role in the cp genomes of the five *Clematis* species. However, only the Ka/Ks of the *ycf1* (*C. nannophylla* and *C. florida*) genes was greater than 1, indicating that the *ycf1* gene was selected for adaptation to the living environment; *ycf1* was also positively selected in previous studies^45^. The *ycf1* gene, the largest gene in the cp and the most potent cp DNA barcode, encodes the ATP-binding (ABC) protein in the cp. *ycf1* is characterised by species specificity^61,70^, rapid mutation rate, and rapid evolution^69^, and has been verified to have classification potential at the subgenus level. In *C. nannophylla,* regions with high purification selectivity were mainly distributed in self-replication (proteins of large ribosomal subunits and subunits of RNA polymerase), photosystem genes (subunits of the photosystem and NADH dehydrogenase), other genes, and unknown genes (*ycf*), similar to the evolution of cp genes in *Pterocarpus*, *Artemisia maritima*, and *Artemisia absinthium*^62,66^, suggesting that strong purification selection preserves specific gene residues and gene functions in these species. Compared to the other four *Clematis* species, the Ka/Ks of *ndhB* of *C. nannophylla* was approximately 0.9, which was significantly higher than that of the other genes. *ndh* is thought to be positively selected for species at relatively high altitudes^71^, which may be due to the higher elevation of the distribution area of *C. nannophylla* compared to the remaining four species of clematis.

### Phylogenetic analysis of the cp genome of *C. nannophylla*

Cp genomes contain a large amount of genetic information that is useful for inferring evolutionary and phylogenetic relationships^72^. Many researchers have used complete cp genome sequences to resolve phylogenetic relationships at various taxonomic levels, and a strong phylogenetic tree can intuitively represent the relatedness of species and evolutionary relationships at various scales. In the present study, we reconstructed a phylogenetic tree with the complete cp genomes of 32 species using the ML method, with six outgroups. The results showed that *C. nannophylla* was more closely related to *C. fruticosa* and *C. songorica* but less closely related to C. *florida*, which is consistent with the results of the classification based on morphological characteristics. *C. nannophylla*, *C. fruticosa*, *C. tomentella,* and *C. songarica* belonged to sect. *Fruticella*, whereas *C. florida* belongs to sect. *Viticella* belongs to the *Clematis* group^6^. The present study also shows that *Clematis* is monophyletic and divides into two large subclades, and *Clematis* forms sister relationship with *Aconitum*^41^.

## Conclusion

In summary, the complete cp genome of *C. nannophylla* was sequenced and compared with those of other related species, providing an important reference for the phylogeny of *C. nannophylla*. Although the cp genomes of *C. nannophylla* were identical to those of other *Clematis* species in terms of genome structure, gene content, and GC content, there were some differences in the boundaries of the IR region. Nucleotide diversity analysis indicated hotspots in the LSC and SSC regions of the cp genes in *C. nannophylla,* which could provide informative markers for the phylogenetic analysis of *C. nannophylla*. Purification selection played an important role in the cp genomes of five *Clematis* species, whereas *ycf1* was positively selective (*C. nannophylla* and *C. florida*). Phylogenetic analysis showed that *C. nannophylla* is closely related to *C. fruticosa*, *C. tomentella*, and *C. songarica*, and the well-resolved phylogenetic tree showed the monophyletic origin of the genera *Clematis* and *Aconitum* as sister genera. The cp genome information obtained in this study provides reference data for molecular marker development, phylogenetic analysis, population studies, and cp genome processing, as well as for better exploitation and utilisation of *C. nannophylla*. These results can guide more efficient germplasm resource utilisation, conservation, and breeding strategies.

### Supplementary Information

Supplementary Tables.

## Data Availability

All annotated chloroplast genomes have been deposited in GenBank (https://www.ncbi.nlm.nih.gov/genbank/), accession number OQ581857.

## Abbreviations

Cp: Chloroplast
LSC: Large single copy
SSC: Small single copy
IR: Inverted repeat
IRa: Inverted repeat a
IRb: Inverted repeat b
SSR: Simple Sequence Repeat
ML: Maximum likelihood
Ka: Non-synonymous substitutions
Ks: Synonymous substitutions
Pi: Nucleotide diversity
